# Report of Deaths in the City of Buffalo, for the Month of June, 1862

**Published:** 1862-08

**Authors:** 


*Report of Deaths in the City of Buffalo, for the Month of June,* 1862.

Accident, 6; Accident by Drowning, 7; Bronchitis, 1; Cancer of the Stomach 1; Cancer of the Breast, 1; Cholera Infantum, 1; Cholera Morbus, 1; Consumption, 11; Convulsions, 17; Croup, 2; Delirium Tremens, 3; Diarrhœa, 2; Disease of rhe Heart, 2; Disease of the Lungs, 2; Disease of the Kidney, 1; Dropsy, G neral, 1; Dropsy Abdominal, 7; Dropsy of the Brain 1; Dysentery, 1; Epilepsy, 1; Erysipelas, 1; Fever, Puerperal, Convulsions, 1; Fever, Scarlet, 7; Fever, Typhoid, 3; Fever, Typus 1; Hemorrhage, 1, Inflammation of the Brain, 4; Inflammation of the Brain and Meninges, 5; Inflammation of the Lungs, 7; Inflammation of the Peritoneum, 2; Marasmus, 2; Measles, 1; Old Age, 5; Scrofula, 1; Small Pox, 1; Suffocation, 1; Sun Stroke, 1; Syphitis; 1; Unknown, 7; Whooping Cough 1; —Total, 118. Ages: 1 and 30 days, 4; 1 month and 6 months, 14; 6 months and 1 year, 10; 1 to 3 years, 15; 3 to 5 years, 12; 5 to 10 years 11; 10 to 20 years, 10; 20 to 30 years, 9; 30 to 40 years, 11; 40 to 50 years, 10; 50 to 60 years, 3; 60 to 70 years, 2; 70 to 80 years, 6; 80 to 90 years, 1; Stillborn, 2; Total, 120. Sex: Males, 68; Females, 51; Not given, 1. Color: White 117; Colored, 2; Unknown, 1. Nativities: United States, 84; German States, 13; Ireland, 13; England, 1; France, 2; Scotland, 1; Canada, 3; Unknown, 3. Parentage: American, 24; German, 42; Irish 30; English, 7; Scotch, 3; French, 2; Prussia, 1; Canada, 1; Unknown, 9. Condition: Married, 19; Single, 82; Widows, 6; Widowers, 2; Unknown, 11. Locality: City at large, 162; Hospital of Sisters of Charity, 9; Buffalo General Hospital, 4; Catholic Foundling Asylum, 4; Small Pox Hospital, 1. By whom certified: by Regular Physicians at Public Institutions, 17; by Regular Physicians in City at large, 43; by Irregular Practitioners, 16; by Coroner, 12; by Undertakers, 32. Total, 120

